# Strand Transfer and Elongation of HIV-1 Reverse Transcription Is Facilitated by Cell Factors In Vitro

**DOI:** 10.1371/journal.pone.0013229

**Published:** 2010-10-06

**Authors:** David Warrilow, Kylie Warren, David Harrich

**Affiliations:** 1 Division of Immunology and Infectious Disease, Queensland Institute of Medical Research, Brisbane, Australia; 2 Griffith Medical Research College, A Joint Program of Griffith University and the Queensland Institute of Medical Research, Herston, Australia; 3 School of Natural Sciences, University of Western Sydney, Hawkesbury, Australia; University of Cambridge, United Kingdom

## Abstract

Recent work suggests a role for multiple host factors in facilitating HIV-1 reverse transcription. Previously, we identified a cellular activity which increases the efficiency of HIV-1 reverse transcription *in vitro*. Here, we describe aspects of the activity which shed light on its function. The cellular factor did not affect synthesis of strong-stop DNA but did improve downstream DNA synthesis. The stimulatory activity was isolated by gel filtration in a single fraction of the exclusion volume. Velocity-gradient purified HIV-1, which was free of detectable RNase activity, showed poor reverse transcription efficiency but was strongly stimulated by partially purified cell proteins. Hence, the cell factor(s) did not inactivate an RNase activity that might degrade the viral genomic RNA and block completion of reverse transcription. Instead, the cell factor(s) enhanced first strand transfer and synthesis of late reverse transcription suggesting it stabilized the reverse transcription complex. The factor did not affect lysis of HIV-1 by Triton X-100 in the endogenous reverse transcription (ERT) system, and ERT reactions with HIV-1 containing capsid mutations, which varied the biochemical stability of viral core structures and impeded reverse transcription in cells, showed no difference in the ability to be stimulated by the cell factor(s) suggesting a lack of involvement of the capsid in the *in vitro* assay. In addition, reverse transcription products were found to be resistant to exogenous DNase I activity when the active fraction was present in the ERT assay. These results indicate that the cell factor(s) may improve reverse transcription by facilitating DNA strand transfer and DNA synthesis. It also had a protective function for the reverse transcription products, but it is unclear if this is related to improved DNA synthesis.

## Introduction

*A priori*, viruses are dependent on their hosts for replication. Therefore, it is not unexpected that recent studies using si/shRNA knockdown [1], [2], [3], [4], [5] and yeast two-hybrid screening implicate host cell factors in HIV reverse transcription [6], [7], [8]. Whether knockdown of the proteins identified in these studies removes a cellular factor which directly supports the reverse transcription complex (RTC) in some manner, or perturbs the general cell environment and ameliorates the replicative functions of the virus machinery though secondary downstream effects is unknown. It is possible that a subset of these factors may act by physical association with the RTC, and evidence of such an association would support a direct role for cell factors. Changes in size and shape of the RTC post-entry suggest this is likely (reviewed in [9]). In the case of yeast two-hybrid studies, some of these cell factors are able to bind one of two key components of the RTC: reverse transcriptase (RT) [7], [8] and integrase (IN) [6], [10], [11] indicating that they may physically associate *in vivo*. Various cell proteins linked to integration have been detected in extracts or partially-purified material containing pre-integration complexes (PICs) [12], [13], [14], [15], [16]. However, similar experiments have not been performed for cell factors implicated in reverse transcription. Therefore, strong evidence for a cell protein which assists reverse transcription as part of a bona fide complex is lacking.

It is possible that cell factors may transiently act on the RTC to facilitate reverse transcription without becoming part of the complex. In the genomic screen performed by Konig *et al*, most of the cell proteins identified to affect reverse transcription were not linked directly to viral proteins but were instead linked to viral RTC components through other protein-protein interactions, suggesting these proteins may be part of large complexes which perform enzymatic functions on the maturing RTC [2]. These complexes function in the ubiquitin-proteasome pathway, DNA transcription, splicing and nucleic acid binding, DNA replication and repair, and nuclear import. Of the more than 50 proteins identified so far to affect reverse transcription, only a few have an activity that suggests a role in assisting reverse transcription (reviewed in [9]). For example, cell proteins with known RNA helicase and DNA repair activities were identified, and these activities might conceivably be related to assisting DNA synthesis during reverse transcription [2]. Some of these proteins might also become part of the RTC as discussed above. So currently, whilst it is likely that cell factors have such a role, none has been convincingly shown either to act on, or be part of, the RTC.

In the search for cell factors which support reverse transcription, *in vitro* biochemical systems provide a powerful means to analyze protein complexes and their activities, which are amenable to further advance by purification of components by chromatographic and other methods. Various *in vitro* reverse transcription systems have been described for retroviruses over the last three decades, mostly based on virion delipidation using high detergent concentration and incorporation of radioactively labelled nucleotides [17], [18], [19], [20], [21]. More recently, a system devised for avian sarcoma and leukosis virus (ASLV) implicated cell factor involvement [22]. Previously, we described an *in vitro* system based on nascent endogenous reverse transcription (ERT) of virions delipidated with low concentrations of non-ionic detergent [23]. The addition of a cytoplasmic lysate prepared from Jurkat cells to an ERT reaction greatly improved the synthesis of late HIV-1 reverse transcription products, also suggesting cell factor involvement. In this work we apply our *in vitro* system to the mechanism of action of the previously described stimulatory cell factor(s). We provide evidence that the strand transfer and elongation ability of the reverse transcription complex released by detergent was improved after addition of lysate. In addition, reverse transcription products were found to be protected from exogenous nuclease when the active fraction was present in the reaction. The above suggest enhanced stability of the RTC by cell factor(s) in the Jurkat lysate *in vitro*, which may assist reverse transcription complex *in vivo*.

## Results

### Improved first-strand transfer contributes to enhanced late product synthesis during endogenous reverse transcription *in vitro*

**Figure 1:**
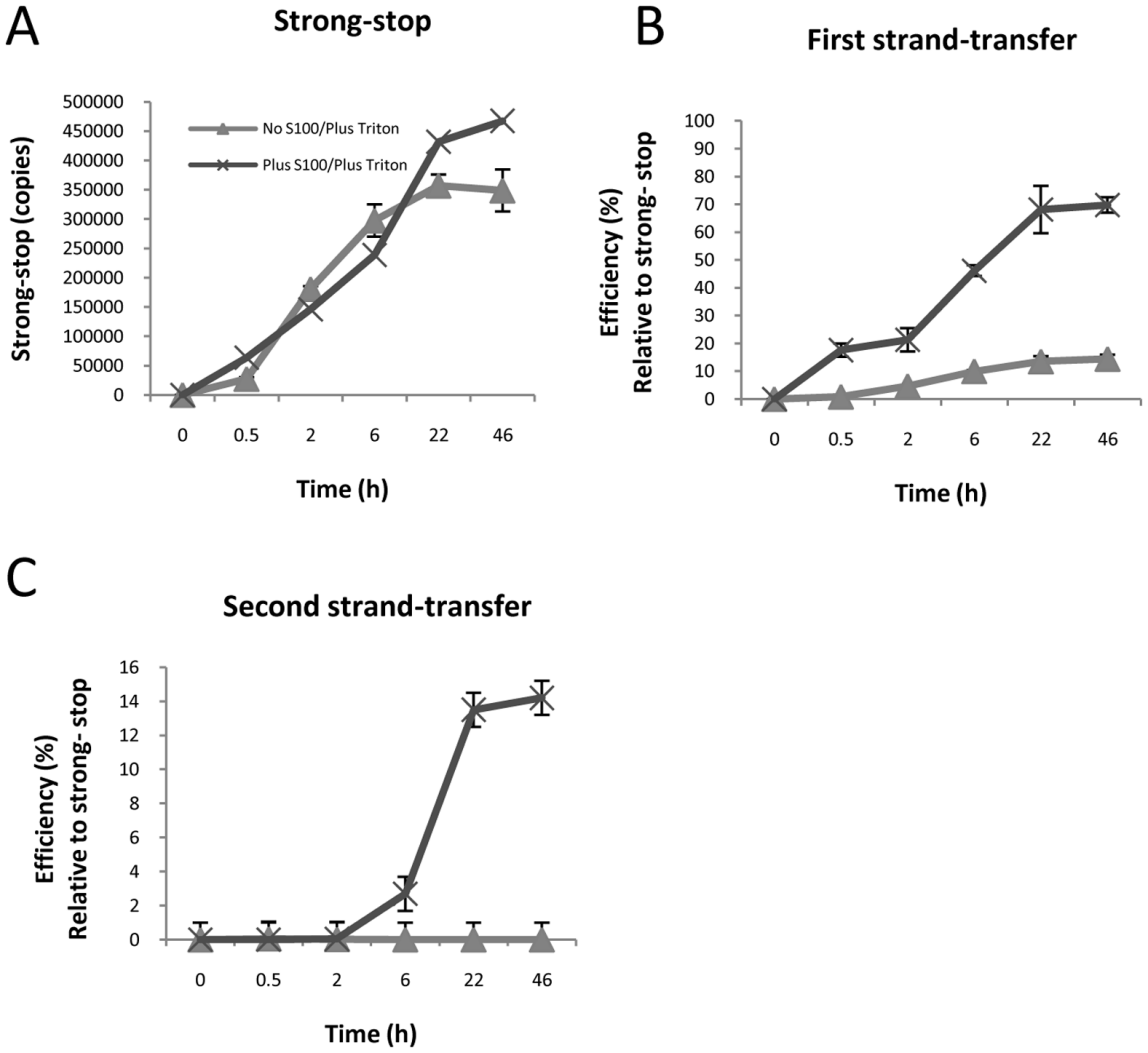
Time course of endogenous reverse transcription (ERT) reactions. Reactions containing detergent-lysed virions were prepared either with or without added S100. Reactions were incubated at 37°C for the indicated time, and reaction products analysed by quantitative PCR using the indicated primer sets (A–C). The experiment was performed in triplicate reactions. The mean value and standard deviation of the mean is shown.

Previously, we showed that an activity in a cytoplasmic lysate (S100) derived from a human T cell line (Jurkat) stimulated the ability of HIV-1 to generate late endogenous reverse transcription (ERT) products *in vitro* in a 22 h reaction [23]. It remains unclear how S100 affects reverse transcription but the possibilities include increasing the efficiency of steps such as strand transfer event or elongation of viral DNA products by RT. To explore this possibility, a time-course of ERT reactions was performed for an extended reaction time of 46 h and multiple products of reverse transcription were measured using quantitative PCR (Fig. 1A–C). The reaction efficiency was calculated which is expressed as the percentage of molecules of products generated for every molecule of negative-strand strong-stop DNA (the first reverse transcription product). The efficiency of late product synthesis in reactions without S100 was not increased using the extended reaction time. As observed previously, second-strand transfer DNA (Fig. 1C), the last measurable DNA product by conventional PCR methods, was greatly stimulated compared to earlier products (strong-stop transfer DNA; Fig. 1A) in the presence of S100. However, first-strand transfer synthesis was improved (Fig. 1B). As the first-strand transfer products precede second-strand transfer products in the synthesis pathway, improved synthesis of first-strand transfer must contribute to the enhanced reaction efficiency of the second-strand transfer.

### The cell factor(s) do not affect detergent lysis of virions

**Figure 2:**
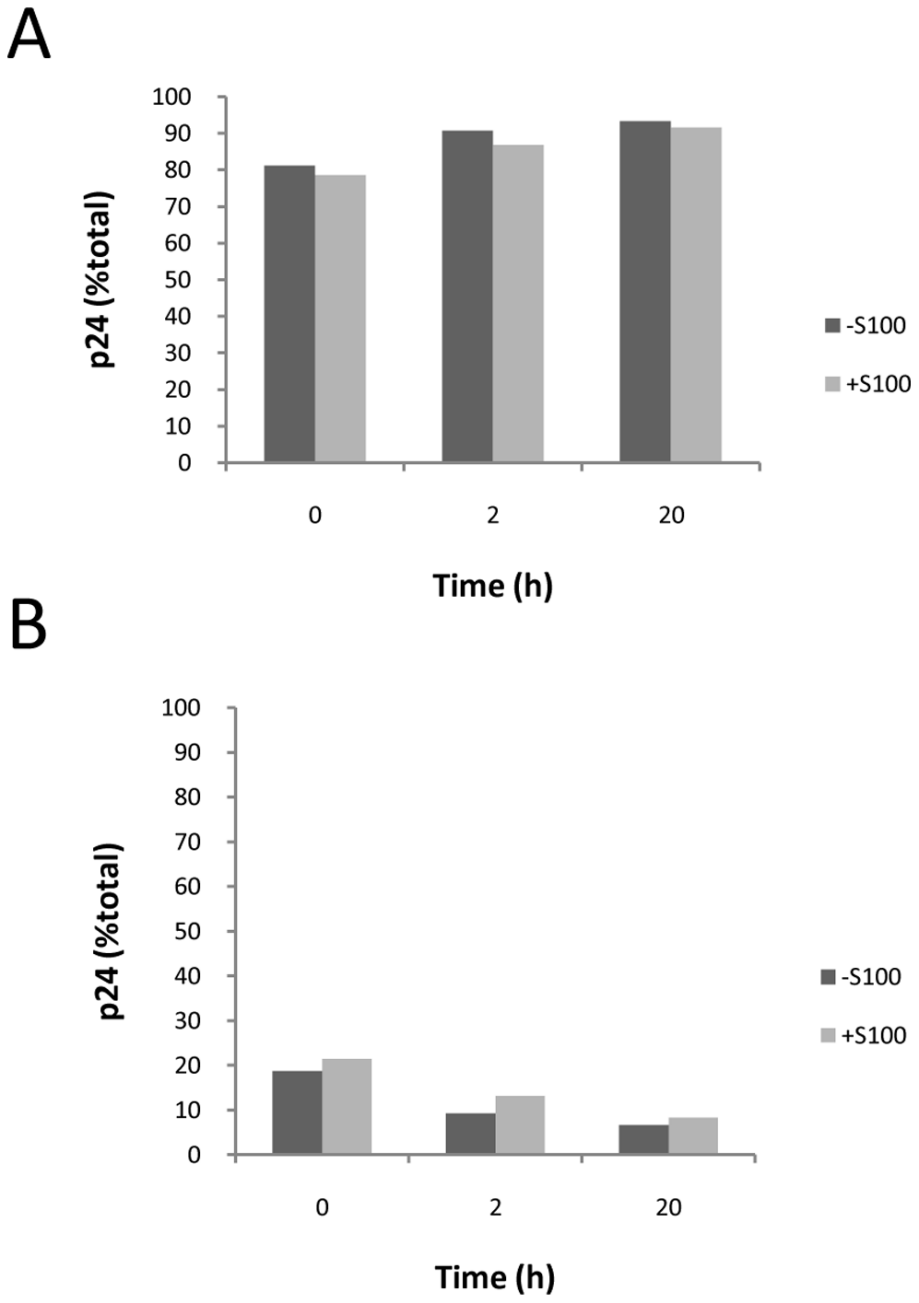
Detergent lysis of virions. Virus was treated with detergent (0.2 mM Triton X-100) in the presence or absence of S100. At the time points shown, samples were subjected to ultracentrifugation, and the amount of p24 in the (A) supernatant and (B) pellet fractions was determined. The data are representative of at least two independent experiments.

The ERT reaction is performed in the presence of detergent at a concentration which we previously demonstrated was sufficient to lyse virions (0.2 mM Triton X-100) [23]. In determining its mechanism of action, an unlikely possibility remained that the cell lysate preparations contained material which retarded the kinetics of virion lysis such that late product synthesis was favoured in the ERT reaction. To test this possibility, virions were treated with detergent (0.2 mM Triton X-100) either with or without S100 for various times up to 20 h. The amount of sedimentable capsid protein remaining, as a measure of unlysed virions and cores [24], was determined by ultracentrifugation and p24 ELISA on the supernatant and pellet fractions (Fig. 2A and B, respectively). Virion lysis was rapid: at least 79% of virions were lysed immediately, as determined by p24 in the supernatant. The amount of p24 in the supernatant increased only slightly with further incubation. This contrasted with the untreated control where >90% of the p24 was in the pellet fraction (data not shown). Triton-X 100 mediated lysis was identical for reactions either with or without S100 which indicated that envelope lysis and capsid core disruption were not affected by addition of cellular factors that stimulated reverse transcription.

### Velocity gradient purification removes a co-purifying RNase activity

**Figure 3:**
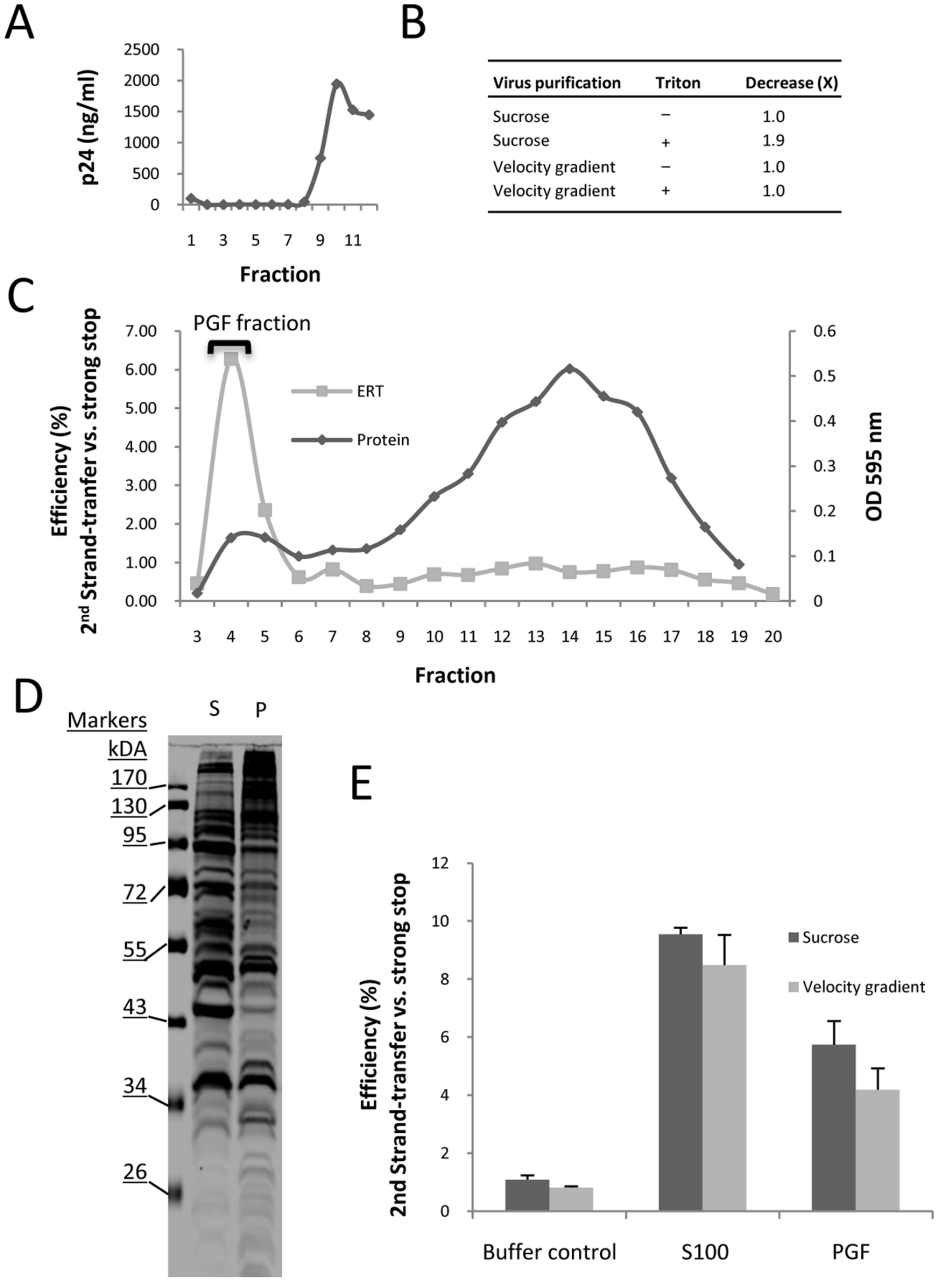
Modification of the ERT reaction components. (A) Sucrose-cushion purified virus was further purified by velocity-gradient ultracentrifugation. (B) The presence of a contaminating RNase was assessed by quantitative RT-PCR on the viral RNA after incubation at 37°C for 1 h. (C) Gel filtration chromatography on the Jurkat cell lysate (S100 fraction). (D) SDS-PAGE on the S100 (S) and peak gel filtration (P) fraction. (E) Comparison of the ability of sucrose-cushion and velocity-gradient purified virus to be stimulated to generate “second-strand transfer” late reverse transcription products by S100, PGF fraction or control lysis buffer. The data shown are representative of at least two independent experiments. The experiment was performed in triplicate reactions. The mean value and standard deviation of the mean is shown.

One possible mechanism of action of the cell factor(s) was that the viral nucleoprotein complex was susceptible to a degradative activity that was prevented by the addition of S100. Northern analysis of viral genomic RNA isolated from lysed HIV-1 indicated that a co-purifying nuclease was present in the sucrose-cushion purified virus preparation (data not shown). Hence, the virus was subjected to further purification by velocity gradient ultracentrifugation [25] to separate the virus from contaminants, such as microvesicles, that might contain nucleases (Fig. 3A and B). A decrease in viral genomic RNA was detected by RT-PCR after addition of Triton X-100 to sucrose-cushion purified virus and incubation at 37°Cfor 1 h, indicating an RNase in this preparation was able to access viral RNA after lysis (Fig. 3B). An RNase activity was not detected in the velocity-gradient purified virus preparation indicating successful purification (Fig 3B). Extension of the assay incubation time did not enable detection of an RNase activity (data not shown). Hence, viral genomic RNA is stable when the highly purified virus is lysed with detergent.

### Reverse transcription efficiency is not improved in highly purified HIV-1 *in vitro*

The stimulatory activity was purified using Sepharose CL-4B gel filtration chromatography (Fig. 3C), which fractionates globular protein with mass between 60,000 and 20,000,000 daltons. The activity was only present in the void volume (the volume of mobile phase in the column) which separated the activity from the bulk of the protein in the S100 whose mobility was retarded by the column matrix. We refer to this activity as the “peak gel filtration” fraction (PGF). SDS-PAGE showed that the PGF had an altered protein profile to the original S100 and an increased proportion of high molecular mass proteins (Fig. 3D). Equal amounts of both sucrose-cushion and velocity gradient purified virus were stimulated several-fold by addition of either S100 or the PGF fraction (Fig. 3E). The PGF gave a slightly reduced late product synthesis compared to S100, which was seen repeatedly, and was probably due to the diluting effects of the gel filtration chromatography on the cell factor(s), but otherwise performed similarly in ERT assays. As we demonstrated that RNase activity had been removed from the purified virus (Fig 3B), poor product synthesis in the ERT reaction after virion lysis could not be attributed to RNase activity. A corollary of this conclusion is that the cell factor(s) do not stimulate late product synthesis by preventing degradation by an RNase present in the virus preparation. Hence, some other activity or effect was responsible for the enhanced late product synthesis when PGF was added.

**Figure 4:**
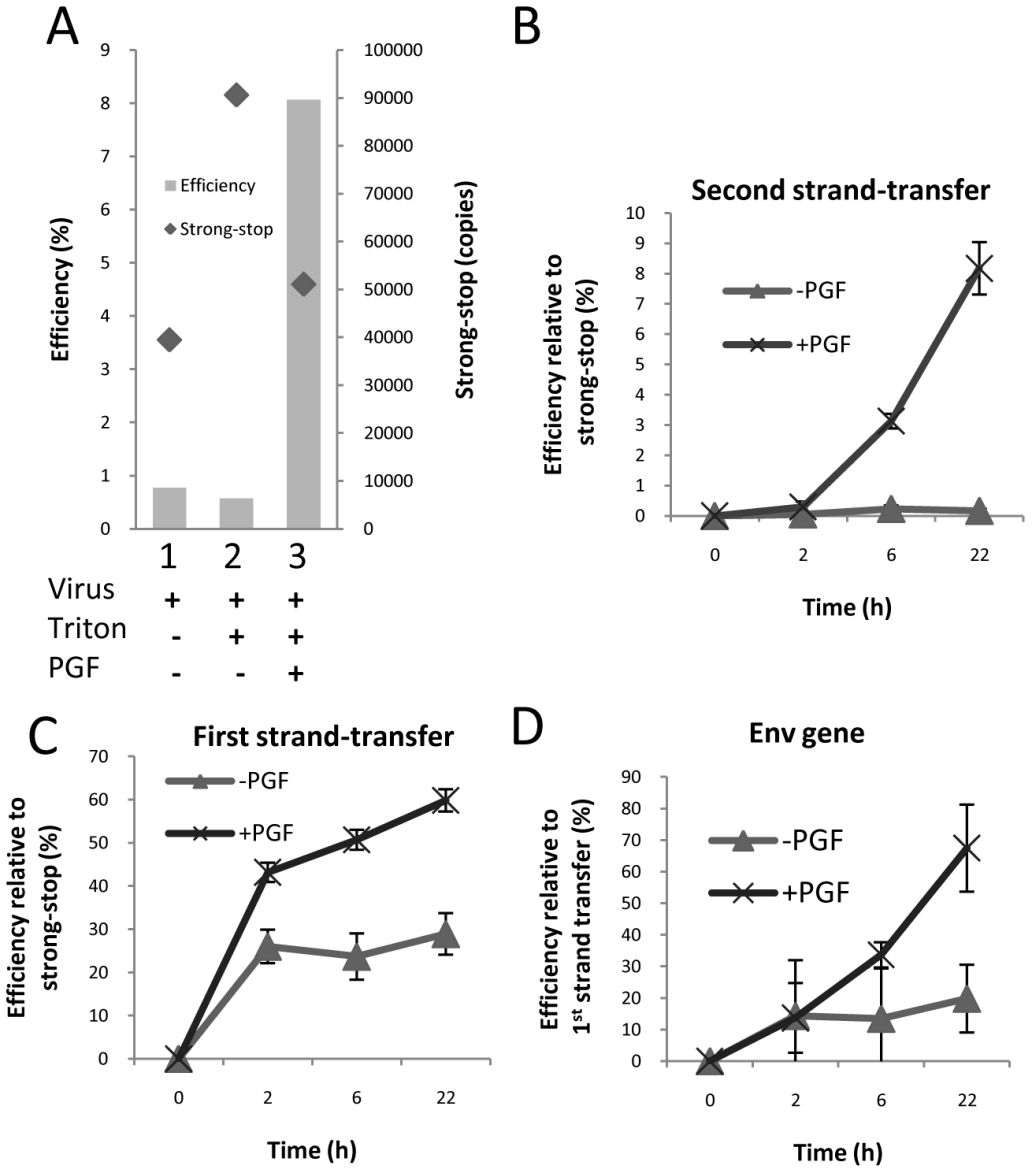
Analysis of modified ERT reaction components. (A) Reaction components were combined as indicated for up to 18 h, followed by ultracentrifugation, resuspension of the pellet, followed by ERT reaction and assay for strong-stop DNA (♦ and right y-axis) and second-strand transfer shown as “efficiency” (gray columns and left y-axis) by quantitative PCR. The data shown are representative of at least two independent experiments. (B–D) ERT reactions were incubated at 37°C for the indicated time, and reaction products analysed by quantitative PCR using the indicated primer sets. For the indicated products, reaction efficiency is given relative to strong-stop (B and C) or first-strand transfer products (D). The experiment was performed in triplicate reactions. The mean value and standard deviation of the mean is shown.

A second hypothesis for a mechanism of action was that the cell factor(s) stabilize the viral nucleoprotein complex after virion lysis. To test this hypothesis, reactions were prepared containing intact virions or detergent-lysed virions either with or without PGF, and they were subjected to ultracentrifugation after incubation at 37°C for 18 h (mimicking conditions used in the ERT assay). The pellet was then resuspended in PBS overnight on ice and an 18 h ERT reaction was performed on the resuspended pellet (Fig. 4A). Detergent-lysed virions in the presence of the PGF were able to generate nascent late product even after incubation at 37°C for 18 h, and a second incubation on ice overnight. The other reactions were considerably diminished in terms of their ability to generate nascent late products. However, all of the reactions made strong-stop DNA indicating that prolonged treatment did not disrupt the viral reverse transcription initiation complex. This suggests that the cell stimulatory factor did not act by stabilizing the nucleoprotein complex with respect to strong-stop DNA synthesis, but enabled efficient reverse transcription following negative strong stop DNA synthesis in agreement with previous experiments.

### Elongation of reverse transcriptase is also stimulated by the cell factor(s)

The contaminating RNase which was present in the original ERT system may have affected the kinetics of reverse transcription product generation. Therefore, we performed additional ERT assays with velocity gradient purified virus was used in ERT reactions over a 22 h period (Fig. 4B–D), in the presence or absence of PGF. The reaction displayed nearly identical kinetics to the original time course experiment with respect to second strand transfer DNA synthesis (Fig. 1B), and these reactions also resulted in a 2-fold increase in first-strand transfer DNA (Fig. 4C).

As late product synthesis was more stimulated than early product synthesis, this suggested that the cell factor(s) may also act by assisting the elongation of DNA by reverse transcriptase. To test this hypothesis, the efficiency of DNA synthesis in the *env* gene region, approximately 2 kb from the U3 region, was determined relative to the first-strand transfer DNA (i.e. measuring the U3-R DNA copy number). Using this product enabled an analysis of elongation without an intervening strand-transfer step. When PGF was present in the reaction, there was a significant increase in the level of *env* gene region DNA relative to first strand transfer DNA (*p*<0.05, *n* = 3) suggesting that elongation of reverse transcription was improved by PGF (Fig. 4D).

### Core stability does not affect cell factor stimulation

**Figure 5:**
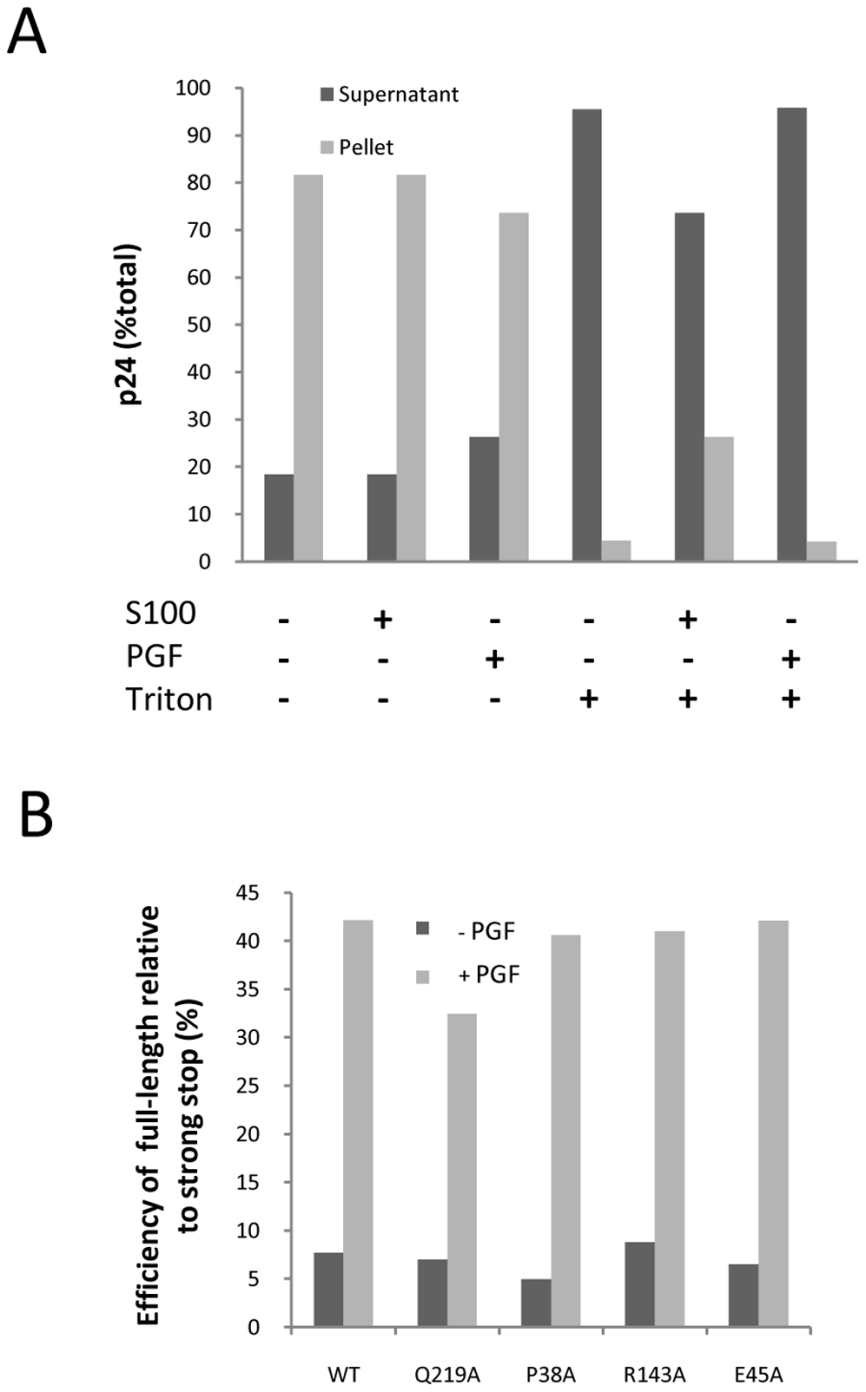
Capsid and endogenous reverse transcription. (A) Virus was lysed with detergent in the presence of S100 or PGF fraction for 18 h, subjected to ultracentrifugation, the pellet was resuspended, and the amount of p24 in the pellet and supernatant fractions was measured. A no-detergent control was also included for comparison. (B) Virus capsid mutants with cores of varying stabilities were used as a source of virus in ERT reactions and their ability to generate late products with and without added PGF fraction was determined by quantitative PCR using the “full-length” primer set. The data shown are representative of at two independent experiments.

We considered that capsid may mediate the effects of the cell stimulatory factor as capsid has been reported in RTCs [26], [27] and plays an important role in infection of non-dividing cells [28]. In addition, capsid was detected in variable but small amounts in purified viral nucleoprotein complexes in our previous study [23]. To further investigate this possibility, we used HIV-1 with CA mutations, which were reported to alter virion core stability [29], in the modified ERT system to determine if reverse transcription was affected by the addition of cell factors. Virion cores with the mutations Q219A, P38A, R143A and E45A were previously determined to be either less or more stable relative to wild-type by the yield of cores purified from detergent-lysed virus (core stabilities: Q219A<P38A<R143A<WT<E45A) [29]. These HIV-1 mutants were defective for reverse transcription in cells but not in a standard ERT assay [29]. Use of the PGF fraction in reactions containing detergent with wild-type virions revealed that lysis was complete, similarly to S100 (Fig. 5A). We then tested mutant viruses for their ability to generate late reverse transcription products either with or without added PGF fraction. As these viruses were derived from cell lines transfected with plasmid, the potential for contamination by plasmid carry-over was greater than the culture-derived virus that was usually used in these reactions. Hence, for these reactions a primer set flanking the major splice donor site (“full-length” primer set) was used for quantitative PCR which increased the signal of nascent product relative to plasmid carried-over from the transfection. Reverse transcription of both wild-type and mutant viruses was stimulated by addition of PGF to the reaction. There was no significant correlation observed between previously determined core stability and late reverse transcription product generation (Fig. 5B). Hence, the CA mutations that affect of changes to core stability *in vitro* and reverse transcription in cells did not affect reverse transcription in the *in vitro* ERT assay containing stimulatory cell factor(s).

### The cell lysate protects nascent reverse transcription product from exogenous DNase I

**Figure 6:**
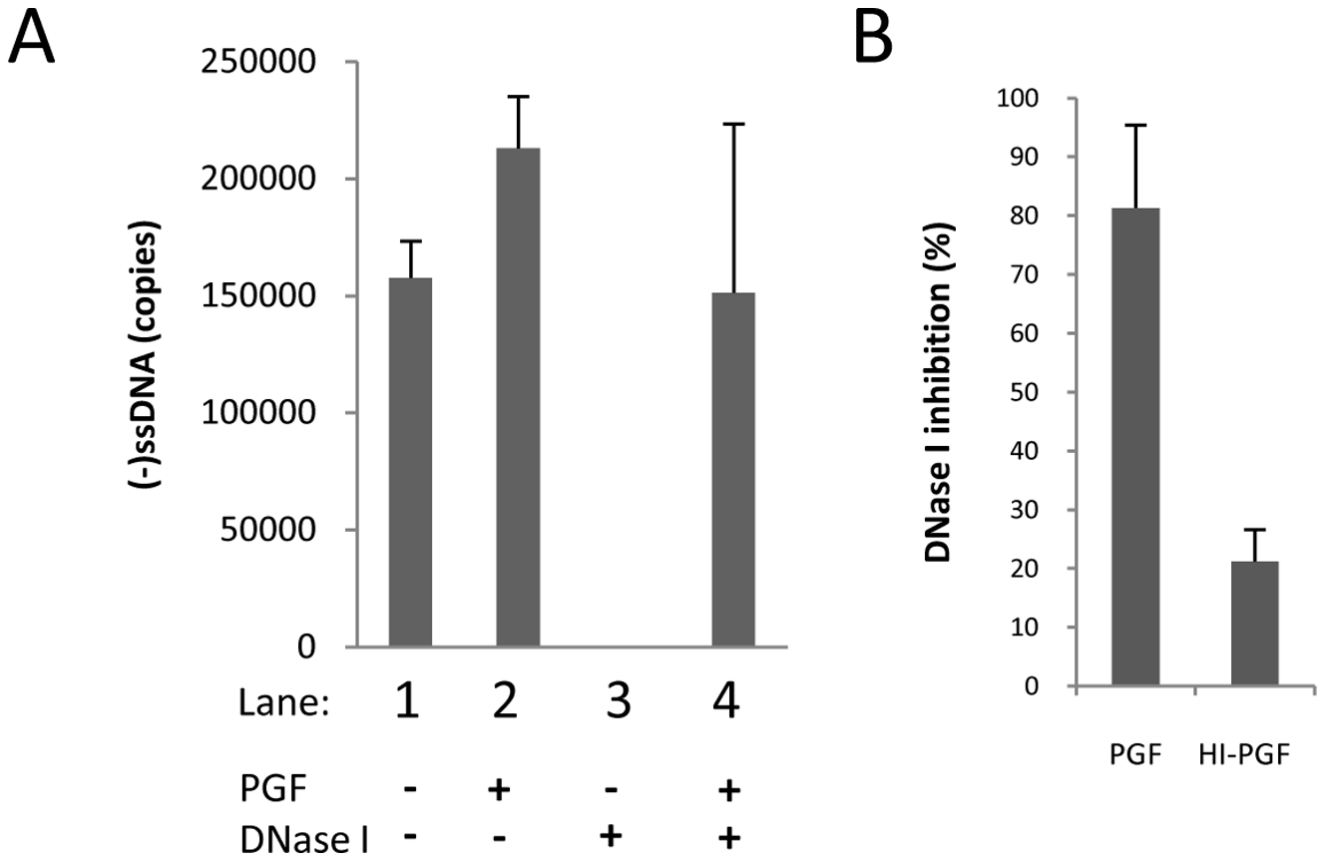
The Jurkat cell lysate contains an inhibitor of DNase I activity. (A) ERT reactions with modified components were prepared as shown with or without exogenous DNase I, incubated 18 h, and strong-stop DNA products determined by real-time PCR. (B) Native (PGF) or heat-denature (HI-PGF) material was assayed for its ability to inhibit DNase I. The data shown are representative of two independent experiments.

It was reported that viral DNA in RTCs and PICs purified from cells were partially resistant to nuclease activity [30], [31], [32]. Our experiments suggested that the PGF fraction affected the viral nucleoprotein complex so that reverse transcription could be completed. The cell factors may have changed the structure of the viral nucleoprotein complex, or acted directly on the viral nucleic acids. To test these hypotheses, ERT reactions were assembled that contained exogenous DNase I, which can degrade single and double strand DNA (Fig. 6), and were incubated for 18 h. The amount of viral DNA remaining was determined by quantitative PCR using primers capable of detecting HIV-1 DNA containing strong-stop sequences (Fig. 6). Nascent HIV-1 DNA was generated in reactions without added DNase I (Fig. 6, reactions 1–2). Reactions prepared with added DNase I and without lysate had no measurable DNA indicating it was degraded (Fig. 6, reaction 3). However, when DNase I was added with PGF, strong-stop DNA was not digested during the overnight reaction (Fig 6, reactions 4). Hence, using the partially-purified reaction components and modified ERT reaction conditions, DNA product was protected from nuclease degradation when the cell factor(s) were present. Following this result, PGF was tested for the presence of an DNase I inhibitor using a fluorometric assay [33]. This assay uses the double-stranded DNA-specific dye PicoGreen to measure a change in fluorescence after DNase I digestion enabling a determination of activity. The PGF fraction (20 µl in a 100 µl reaction) inhibited DNA degradation by approximately 80%. Interestingly, heat inactivation of the PGF fraction at 80°C for 15 min reduced this inhibitory activity to approximately 20% (Fig. 6B). Heat inactivation of the inhibitor suggests a proteinaceous activity, and is co-incidental with the heat-labile nature of the cell stimulatory factor [23]. It's unclear whether PGF protected viral nucleic acids from DNase I activity directly or inhibited the nuclease. In either case, this appears to be a general feature of the cell factor(s) as viral factors were not required (Fig. 6B).

## Discussion

Many of the post-entry events that regulate reverse transcription of HIV-1 genomic RNA remain unresolved. Some of these events most likely involve host cell proteins, and reverse transcription has been linked to several cell factors (reviewed in [9], [34]). We previously identified an activity in Jurkat cells which stimulates the synthesis of late reverse transcription products. In this study we have taken steps to further define how this factor acts in our ERT system. Further purification of components of our *in vitro* system has enabled us to eliminate a contaminating RNase activity as being responsible for poor late product synthesis in lysed virions. Instead, addition of cell factor(s) significantly improved first strand transfer and late product synthesis of reverse transcription, most probably by improving DNA elongation by RT (Figs. 2 and 3). Cell factors did not appear to enhance the stability of the viral nucleoprotein complex initiation complex (Figs. 1 and 3A), but could protect viral DNA from degradation by exogenous nucleases (Fig. 6).

We eliminated a number of possible mechanisms by which the cell factor(s) may improve late product synthesis. The S100 Jurkat cell lysate stimulated the lysed virions to complete reverse transcription rather than affected the ability to initiate reverse transcription. Previously we had demonstrated lysis of the viral membrane when 0.2 mM Triton X-100 was added [23]. Here we speculated that virion lysis was altered such that the lysate reduced the effective concentration of Triton X-100 in the reaction and this somehow assisted reverse transcription, favouring DNA product completion. However, we showed that virion lysis was rapid and there was no substantial temporal difference in the ability of Triton-X100 to liberate soluble CA in the presence or absence of S100 (Fig. 2). In addition, mutations in CA that affect virion core stability *in vitro,* and down regulate reverse transcription in cells, did not change the degree to which the wild type or mutant virus could be stimulated by cell factor(s) (Fig. 5.). Hence, these results do not support a role for capsid in mediating the stimulatory effects of the cell factor(s) in our *in vitro* system. But this does not excluded a role for capsid in reverse transcription *in vivo*.

To advance our insight of the mechanism of improved ERT by cell factors, both the virus and stimulatory factor were subjected to further purification. We used a 6–18% iodixanol gradient to purify HIV-1 particles away from microvesicles which are known to contain a various cell proteins and activities [25] to produce a highly purified virus preparation. Velocity gradient purification resulted in a reduction in a contaminating RNase activity (Fig. 3). In combination with gel filtration-purified stimulatory factor from the S100, where the stimulatory factor was present exclusively in the column void volume (the PGF fraction), viral RNA was relatively stable post-lysis in the presence of reaction components. In spite of these substantial improvements to the ERT system, the efficiency of late reverse transcription products by the viral nucleoprotein complex remained low unless S100 or PGF fraction was added to the reaction (Fig. 3). This indicated that the relatively poor late product synthesis of detergent-lysed virions was not due to the actions of an RNase activity; therefore, the lysate does not act by neutralizing such an activity. Instead, the cell factor(s) stimulates late product synthesis by some other means. We observed that first strand-transfer process was completed in 2 h without PGF, whereas first strand-transfer copies increased if PGF was present (Fig. 4C). When we measured DNA levels in the *env* region located approximately 2 kb from the first strand-transfer region, comparison of DNA levels a marked increase in DNA copies in *env* region between 2 and 22 h if PGF was present (i.e. after the completion of first strand-transfer) suggesting that PGF improved DNA elongation by RT (Fig. 4D). Further experiments will be required to reveal how PGF affects RT interaction with the primer-template.

Addition of S100 or PGF fraction to the ERT reaction in the presence of exogenous DNase I protected the DNA product from digestion. How this observation relates to the ability to stimulate late product synthesis is currently unclear. The cell factor(s) may change the biochemical properties of the viral nucleoprotein complex such that it becomes nuclease resistant. It may bind nucleic acids promoting reverse transcription DNA strand transfer or elongation resulting in enhanced late product synthesis, or it could somehow inactivate the nuclease activity (Fig. 6). In doing so it may protect the DNA product from an exogenous nuclease. While it is entirely possible that the observation of nuclease resistant viral DNA in this study may be co-incidental, RTCs isolated from infected cells make viral DNA that is resistant to degradation by nucleases [30], [31], [32], making it possible that PGF contains cell factor(s) relevant to RTCs purified from cells.

The stimulatory activity eluted in the void volume of a number of different gel filtration media. We routinely used CL-4B medium for purification in which globular molecules greater than 20 MDa would be expected in the void volume. This suggests that the stimulatory activity may be associated with a large complex or that may be simply binding fragmented DNA present in the S100. Regarding the former, some of the cell proteins linked to HIV-1 reverse transcription by a siRNA genomic screen were components of large interacting networks suggesting multi-protein complexes [2]. Some of these proteins were also nucleic acid binding proteins or involved in DNA repair which may potentially protect a DNA product from a nuclease activity. The latter possibility, association with a DNA fragment, is also consistent with a DNA binding protein. We attempted to remove DNA and RNA from the S100 using micrococcal nucease before gel filtration, but this treatment did not shift the elution profile of PGF activity (data not shown). We cannot exclude the possibility that nuclease resistant DNA was present in the S100. We are currently in the process of purifying the cell factor(s), and its identification should elucidate its composition and the means by which it enhances reverse transcription in HIV-1 *in vitro*. The next step will then be to determine whether it is biologically relevant to early infection *in vivo*.

In conclusion, endogenous reverse transcription could be strongly stimulated by the addition of partially purified cell factor(s) that had two notable effects: increased ability to undergo first strand transfer and elongation that could account for the increase in late products, and the viral DNA became resistant to exogenous DNase I activity. Further purification will be required to identify the cellular proteins that responsible, so that their relevance can be determined in the context of cell infection.

## Methods

### Cell lines and virus culture

All cells were grown in RPMI1640 supplemented with 10% NBS and penicillin-streptomycin at 37°C in 5% CO_2_ (standard conditions). The MAGI CXCR4-expressing cell line (NIH AIDS Research and Reference Reagent Program) was additionally supplemented with 0.2 mg/ml geneticin and 0.1 mg/ml hygromycin B. Stocks of HIV_NL4.3_ (National Institutes of Health AIDS Research and Reference Reagent Program) or HIV-1 containing capsid mutations (a kind gift of Christoper Aiken) were generated by transfection of the corresponding proviral DNA using Lipofectamine™ 2000 (Invitrogen) or FuGENE6 (Roche) into HEK293T cells (a gift from Dr. Richard Gaynor, Eli Lilly and Co., USA) according to the manufacturer's recommendations. After 4 h incubation, the transfection mix was removed and the cells were washed with fresh medium 4 times. Standard growth medium was then added. Cell culture supernatants were removed at 48 or 72 h post-transfection, centrifuged (200 *g*, 10 min), and the supernatant was filtered (0.45 µm) and stored in 1 ml aliquots at −80°C. Amplification of the HIV_NL4.3_ stock in the MAGI cell line was as described previously [23]. Briefly, the cells were infected with HIV_NL4-3_ (250 ng p24), incubated for 6 days, harvested and stored as above. Virus was concentrated by ultracentrifugation (Beckman Coulter Sw41Ti, 100000 *g*, 2 h, 4°C) on a 20% sucrose cushion. If the virus was from a stock generated by transfection without prior amplification in MAGI cells, ultracentrifugation was followed by a 1 ml wash in PBS. The pellet was resuspended overnight in sterile PBS in one-tenth of the original culture volume, and stored in 50 µl aliquots at −80°C until needed. The concentrated virus stock was further purified by velocity gradient ultracentrifugation as in Cantin *et al*. (2008) [25] using Optiprep (Axis-Shield) diluted from 6% to 18% in eleven 1.2% steps in PBS buffer. Optiprep was added in layers of 820 µl, starting with the 18% layer at the bottom and progressively decreasing to 6% and centrifuged at 100,000 *g* for 3 h (Beckman Coulter Sw41Ti, 28000 rpm, 4°C). Fractions (820 µl) were collected from the top of the tube. The fractions were assayed for p24 antigen and the peak fractions were pooled and stored in aliquots at −80°C.

### ERT assays

Endogenous reverse transcription (ERT) reactions were performed by addition of virus (equivalent to 10 ng p24 unless otherwise stated) to a mixture containing 10 mM Tris, pH 7.4, 10 mM MgCl_2_, 200 µM each dNTP and 0.2 mM Triton X-100 in nuclease-free water (final volume of 50 µl) for 18–20 h at 37°C. Detergent was the last component added. A no-nucleotide control reaction was always included and was negligible or the data were discarded. If relevant, the amount of S100 or PGF fraction added varied but was typically 20 µg or 1.5 µg, respectively. Reactions products were extracted, once with an equal phenol:chloroform:iso-amyl alcohol (25∶24∶1) and once with chloroform. The extracts were ethanol precipitated, washed with 70% ethanol, dried and resuspended in 100 µl of 0.1 mM EDTA. Purified reaction products (5 µl) were added to the reaction mix containing 0.4 µM of each primer, SYBR Green I, 30 U/ml Platinum Taq polymerase, 20 mM Tris-HCl pH 8.4, 50 mM KCl, 3 mM MgCl_2_, 200 µM each dNTP, 20 U/l uracil-N-glycosylase (Invitrogen) in a final volume of 15 µl. When reactions contained DNase I it was added at 500 U/ml. A no-DNA control (5 µl of 0.1 mM EDTA, pH 8.0) was also included. Standard primer sets used for amplification were: strong-stop DNA, forward primer (5′-dGGTCTCTCTGGTTAGACCA-3′) and reverse primer (5′-dAAGCAGTGGGTTCCCTAGTTAG-3′) and; first-strand transfer, forward primer (5′-dAGCAGCTGCTTTTTGCCTGTACT-3′) and reverse primer (5′-dACACAACAGACGGGCACACAC-3′); full-length minus strand, forward primer (5′-dCAAGTAGTGTGTGCCCGTCTGTT-3′) and reverse primer (5′-dCCTGCGTCGAGAGAGCTCCTCTGG-3′); second-strand transfer forward primer (5′-dAGCAGCTGCTTTTTGCCTGTACT) and reverse primer (5′-dCCTGCGTCGAGAGAGCTCCTCTGG-3′); and envelope forward primer (5′-dGGTCCGAGATCTTCAGACCT-3′) and reverse primer (5′-dGTGGGTGCTACTCCTAATGG-3′). The mixes were subjected to cycling: [50°C, 2 min; 95°C, 2 min]_1_[95°C, 15 sec; 65°C, 30 sec]_40_ on a Rotor-Gene 3000™ thermocycler (Corbett) set to collect SYBR fluorescent signal after the 65°C step. Copy number (proviral equivalents) was determined by reference to a standard curve prepared by dilution of plasmid pNL4.3.

### Detergent treatment and p24 assay

Triton X-100 detergent was added at room temperature to virions at the concentrations indicated in the text. The samples were then immediately diluted to 10 ml in 0.1 mg/ml BSA dissolved in PBS and centrifuged at 100,000 *g* for 2 h (Beckman Sw41Ti rotor, 28000 rpm, 4°C). An untreated virus control, which was harvested at 0 h, was included. Pre-centrifugation, supernatant and pellet fractions were assayed by ERT or for p24 antigen using RETROtek HIV-1 p24 antigen ELISA (Zeptometrix) according to the manufacturer's instructions.

### Preparation of Jurkat cell lysates and gel filtration

Preparation of a cleared lysate from Jurkat cells (S100) was as described previously [23]. All buffers contained protease inhibitor cocktail (Roche). Cells grown to near-confluence were washed and lysed using the Dounce homogeniser. The lysate was cleared by centrifugation in a refrigerated microfuge (4°C, 12000 rpm, 10 min) followed by ultracentrifugation at 100000 *g* for 1 h (Beckman Coulter Sw41Ti rotor, 28000 rpm, 4°C). The S100 fraction was partially purified by gel filtration chromatography using CL-4B medium (Sigma). The fractions were assayed by ERT as above and the majority of the activity resided in the void volume material as determined by co-fractionation with chromosomal DNA. Protein concentration was measured using a commercially available Bradford assay (Bio-Rad, CA, USA). The final protein concentration varied from preparation to preparation but was typically 4 mg/ml for S100 lysate and 0.15 mg/ml for the void volume fraction.

### Genomic viral RNA assay

Viral RNA stability was measured by combining the ERT reaction components at 37°C for 1 h. Reactions were extracted as for the ERT reaction above, resuspended in 20 µl of 0.1 mM EDTA. cDNA synthesis was performed on the extracted RNA using Superscript III (Invitrogen) and primer (5′-dACACAACAGACGGGCACACAC -3′) according to the manufacturer's recommendations at 37°C for 45 min then the reaction was terminated by heating at 75°C for 15 min. The cDNA was diluted 1∶10 and used as a template for the quantitative detection of strong-stop DNA as for the conditions of the ERT assay using forward and reverse primers as above.

### PicoGreen DNase I assay

Determination of DNase I activity was as described by Choi and Szoka [33]. The reaction contained the sample, 50 pg DNase I, herring-sperm DNA (0.2 µg) in a reaction containing 25 mM HEPES pH 7.4, 4 mM CaCl_2_, 4 mM MgCl_2_ in a final volume of 100 µl. The reaction was incubated for 30 min at 37°C. To detect double-stranded DNA, a concentrated stock of PicoGreen was diluted 1∶200 and added to the reaction mixture (100 µl). The fluorescence intensity was determined by excitation at 485 nm and detection at 520 nm. A heat-inactivation control was prepared by heating the PGF fraction at 80°C for 15 min. The change in fluorescence was determined by subtracting the fluorescence of the sample from the excitation fluorescence of a no-DNase I control. In the case of the PGF fraction samples, the excitation fluorescence was subtracted from a control containing the same sample contents but without DNase I. The DNase I activity was then determined with reference to a standard curve (0–50 pg DNase I).

### Gel electrophoresis

Protein samples were subjected to electrophoresis by 10% SDS-PAGE according to Sambrook *et al* [35]. Proteins were detected by using Bio-Safe Coomassie stain (Bio-Rad) according to the manufacturer's instructions.
